# Anemia and its determinant of in-school adolescent girls from rural Ethiopia: a school based cross-sectional study

**DOI:** 10.1186/s12905-019-0791-5

**Published:** 2019-07-17

**Authors:** Rediet Takele Regasa, Jemal Ali Haidar

**Affiliations:** 1 School of Public Health, Wollega University, Wollega, Ethiopia; 2 School of Public Health, Addis Ababa University, Addis Ababa, Ethiopia

**Keywords:** Adolescent, Anemia, Determinants, Ethiopia, Thinness, Wayu Tuqa

## Abstract

**Background:**

The rapid growth and changes that occur in adolescents increase the demand for macro and micronutrients and addressing their needs particularly in females would be an important step to break the vicious cycle of intergenerational malnutrition. Thus we evaluated the status of anemia and its anthropometric, dietary and socio demographic determinants in female adolescents, west Ethiopia.

**Methods:**

A school based cross-sectional study was conducted among school going adolescent girls of Wayu Tuqa district, south west Ethiopia and a 3-stage random sampling technique was used to select study participants. Data were entered into EpiData version 3.1 and analyzed using STATA version12. Haemoglobin was measured by HemoCue 301+ photometer and WHO Anthro-plus software Version 1.0.4 was used to calculate BMI for age z-score. Both bivariate and multivariate analyses were performed to check associations and control confounding. A *p*-value <0.05 was considered statistically.

**Result:**

The overall prevalence of anemia was 27% (95% CI: 22.9–31%) of which 23, and 4% had mild and moderate anemia respectively**.** The proportion of thinness and overweight girls based on the BMI for age z-score was 33 and 3.6%, respectively. The odds of developing anemia were almost four times more likely among late adolescents as compared to early adolescents (AOR = 3.8 95%CI = 2.3 to 8.5).Adolescents from rural areas were 3.4 times more likely to have anemia as compared to their urban counterparts (AOR = 3.4 95%CI = 1.9 to7) and adolescents those who attained menarche were two times more likely to develop anemia compared to those who did not attained menarche (AOR = 2.3 95%CI = 1.34 to 4.2).

**Conclusion:**

The prevalence of anemia among adolescent girls was a moderate public health problem. To improve the prevailing nutritional problem, there must be inter-sectorial collaboration among health sectors and education sectors in providing nutritional education and counseling based on age and menarche status.

**Electronic supplementary material:**

The online version of this article (10.1186/s12905-019-0791-5) contains supplementary material, which is available to authorized users.

## Background

Anemia is one of the most common nutritional deficiency disorders which is a serious global public health problem leading to low birth weight including morbidities and mortalities of mothers and children in addition to negative consequences on the cognitive and physical development of children, and poor productivity in adults [1]. According to the 2008 global database report of the World Health Organization, anemia is affecting over 1.62 billion individuals [2]. Despite a significant global reduction of anemia in the past two decades, the problem still remained a prime cause of disability in women and young children [3, 4] with some variations across developed and developing countries [5]. The prevalence varied considerably by socio-demographic/economic status and physiological status of women population [6–9].

The most vulnerable population groups to anemia are women of reproductive age groups particularly during pregnancy and lactation due to their increased demand for iron and other important nutrients [10]. Other important at risk groups are adolescents whose nutrient requirement increases during this period as a result of the rapid changes occurring in their physical dimension and body composition [11]. Since this period is often overlooked, adolescents are exposed to several forms of macro and micronutrient malnutrition [11, 12] and thus the period is the last opportunity to break the vicious cycle of intergenerational link of the nutritional problem [13, 14]. While the WHO estimate of global anemia among pregnant women and non-pregnant women aged 15–49 years is 42 and 30% respectively, the gap is wider in developed and developing countries [8]. The prevalence of anemia is 14% in developed and 51% in sub Saharan countries [5, 15, 16] of which half of the anemic cases are female adolescents [13].

The prevalence of anemia in Ethiopia on the other hand varied and ranged from 44 to 9% with some moderate variation by urban-rural residence and region based on the recent Ethiopian demographic health survey (EDHS) [14]. Although the EDHS report availed the overall magnitude of anemia, it is lacking the disaggregate information of adolescents demonstrating a gap of information. Other recent studies conducted in the country on anemia are either based on small-scale field or hospital surveys with limited geographical scope and coverage [14, 17, 18] indicating the available information in this segment of population is inadequate.

This study has therefore addressed the identified gap and evaluated the status of anemia and its anthropometric, dietary and socio demographic determinants in Ethiopian female adolescents for some program initiatives in the country.

## Methods

### Study area and population

A school based cross-sectional study was conducted among in-school adolescents aged 10–19 years from February to March 2016 in ***Wayu Tuqa*** district of East ***Wollega*** Zone, ***Oromia*** Regional state, Ethiopia. The district is 316 km east west of Addis Ababa and has twenty-six primary and two secondary governmental schools. Topographically, the district constitutes of highland, Midland, and lowland with an elevation ranged from 1729 to 2740 m above sea level. The district was conveniently selected because of the unavailability of nutritional information particularly on anemia which the principal author felt a major gap and contribute to the school community which she is very familiar.

### Sample size determination and sampling procedure

The sample size was estimated using a single proportion formula based on the national prevalence of anemia of 25% among late adolescent groups [19] with 95% confidence level and 5% margin of error which was further inflated by a design effect of 1.5 and 5% non-response rate with a final samples of 454. Two-stage random sampling techniques were applied to select the sampled adolescent. The first-stage units were schools and the second-stage units were the school girls. Prior to allocation of the schools and the participants, information on the number and size of schools in the study area was obtained from the district education authority to constitute the sampling frame. Then the schools were ranked by geographical location to allow for equal distribution of the schools over the study area and then selected randomly. From the prepared sampling frame, the estimated number of participants was proportionally allocated to allow for equal distribution over primary, secondary and preparatory schools. Accordingly 72, 241 and 135 sampled adolescents from highland, midland and lowland, respectively were allocated proportionately. Finally the participants were selected by systematic random sampling technique with the first case being selected randomly.

### Data collection

Data were collected using a pre-tested interviewer administered questionnaire which included information on socio-demographic, reproductive health status (age of menarche), history of anthropometric measurements, hemoglobin status and food intake (Additional file 1). Food intake was assessed by using dietary diversity score (DDS) qualitatively based on nine food groups comprised of starch (cereals, and roots), vegetables, fruits, fish and tubers, meat (including organ meat), milk, egg and legumes consumed in twenty four hours using recall method. Each food group was then counted only once resulting in a possible score of 0 to 9. Since there is no international consensus on which food groups to include in the scores to create dietary diversity scores, we categorized the score as low DDS, medium DDS and high DDS when the food consumed were less than or equal to 3, 4–6 and above 6 food groups, respectively.

Prior to data collection, 2 days intensive training (objective of the study, interviewing approach, blood specimen collection, and confidentiality) was given to six diploma nurses recruited as data collectors by the principal author.

#### Height

Was measured using a wooden length measuring board with a sliding head bar noted to the nearest 0.1 cm following standard procedure in Frankfurt plane without shoes.

#### Weight

Was measured using a battery powered electronic personal weighing scale to the nearest 0.1 kg with minimum clothing and with no footwear. The scale was checked against known weight regularly and all measurements were taken twice and the average was computed. To avoid variability, the same measurer was assigned among the nurses to do all the anthropometric measurements.

#### Sample collection

Hemoglobin was measured at the spot using a battery-operated portable HemoCue hg 301 + Analyser. Capillary blood samples was drawn aseptically from the respondents’ finger by a micro cuvette and read immediately.

The geographic locations and elevations of visited schools were determined by using a hand held Global Positioning System (GPS) (Garmin GPSMAP®).

### Data quality control

Data quality was insured by training data collectors as well by providing day to day supervision during the entire data collection period. Pre-test and demonstration of the tools were done among 5% of non-sampled adolescents.

The collected data were checked for their completeness, clarity and consistency by the principal author on daily basis. Reliability and precision of the anthropometric scales and HemoCue machine were also checked against the standard regularly.

### Data analysis and statistical test

Data were entered into Epi-data version 3.1 and then cleaned, coded and analyzed by stata version12. Anthropometric data were entered and analyzed using WHO Anthro-plus version 1.0.4.software. Descriptive summary (Frequency distribution, proportion, mean & standard deviation) was used to summarize the variable. Bivariate & multivariate logistic regression was used to see the association of anemia with socio-demographic, dietary and other important factors by calculating odds ratios and 95% confidence intervals. A *p*-value less than 5% was taken as statistically significant.

WHO-anthro-plus software version 1.0.4 was used to compute BMI for age z-scores (BAZ) to classify the nutritional status of the adolescents. Adolescents were classified as underweight or thin when BAZ was <−2SD and overweight/obese (> + 1D).

Hemoglobin was classified as normal (hg > 12 g\dl, mild (10–11.9 g\dl), moderate (7.0–9.9 g/dl,) or severe (<7 g\dl) based on the WHO recommended cut off points after adjustment for altitude was made. The principal components analysis was used to determine household asset quintile of all respondents.

## Result

A total of 448 adolescents were enrolled with response rate of 98.0%. Over half (53.8%) were from midland topography and 249 (55.6%) were aged between 15 and 19, and the mean age was 14 + 2 years. Three quarter (75%) of them were from rural and the vast majorities (96.2%) were single. Protestants constituted 341 (76.1%) and nearly all (97.1%) were from Oromo ethnicity. About two-thirds (65.6%) were from grade nine and 331(73%) from family members of five and above. Over half (54.2%) of respondents’ father and 207(46.2%) of respondents’ mother had no formal education. The proportion of poorest, poor, middle, rich and richest wealth quintile category across the respondents was almost uniform (Table 1).

**Table 1:** Demographic and socio-economic characteristics of respondents in *Wayu Tuqa* district Ethiopia, 2016 (*n* = 448)

Characteristics	Levels	Frequency (n)	Percent
Residence	Urban	112	25
	Rural	336	75
Agro ecology	Highland	72	16
	Midland	241	53.8
	Lowland	135	30
Age	10–14	199	44.4
	15–19	249	55.6
Marital status	Single	431	96.2
	Ever married	17	3.8
Grade	Primary	294	65.6
	Secondary	83	18.5
	Preparatory	71	15.9
Family size	1–5	117	26.1
	> 5	331	73.9
Fathers education	None	243	54.2
	Primary	100	22.3
	Secondary	105	23.4
Mothers education	None	207	46.2
	Primary	131	29.2
	Secondary	110	24.6
Wealth quintile	Poorest	88	19.7
	Poor	91	20.4
	Middle	90	20.1
	Rich	89	19.9
	Richest	89	19.9
Total		448	100

### Reproductive health characteristics of respondents

About two-thirds (67.2%) of participants had attained menarche and more than half 168 (54.2%) attained their menarche before the age of thirteen with mean age of 13.3 ± 1.2.More than half (60%) of them had bleeding duration of less than 5 days (Table 2).

**Table 2:** Reproductive health information of respondents in *Wayu Tuqa* district Ethiopia, 2016

Characteristics	Frequency (n)	Percent (%)
Attained menarche		
Yes	301	67.2
No	147	32.8
Age at onset of menses (in years)		
≤13	168	37.5
>13	133	29.7
Duration of bleeding (in days)		
<5	180	60
>=5	121	40
Total	448	100

### Anthropometric measurements among respondents

BMI for age z-core (BAZ) was used to determine the respondent’s nutritional status and nearly two-thirds (62.0%) had normal BAZ status. Adolescents with thin BAZ status constituted 147.8(33%), while overweigh (+ 1 SD and + 2 SD) and obese (> + 2SD) were 16(3.6%) and 6 (1.4%), respectively.

### Magnitude and severity of anemia

The mean hemoglobin level of adolescent girls was 12.6 ± 1.4, which ranged from 6 to 14.6 g/dl. The overall prevalence of anemia was 121 (27%). The proportion of mild and moderate anemia was 103 (23%) and 18(4%) respectively.

### Dietary diversity score (DDS)

Nearly all (99.3%) participants consumed starch staples. The proportion of respondents who consumed vegetables, fruits, tuber, meat, eggs, legumes and milk within 24 h prior to the study were 19.2, 23.2, 53.8, 16.7, 12.9, 54.5 and 50.5%, respectively indicating consumption of meat, which is good source of bio-available iron, was low.

The mean DDS was 3.3 + 1.24. Over half (56%) of the adolescent girls had low DDS and the rest 183(41%) and 12(3%), had medium and high DDS, respectively (Table 3).

**Table 3:** Food groups consumed by participant (*n* = 448) 24 h prior to the survey in *WayuTuqa* district, Ethiopia, 2016

Food group	Frequency(n)	Percent (%)
Starchy staples ^a^	445	99
Vegetable	86	19.2
Fruit	104	23.2
Tuber	241	53.8
Meat	75	16.7
Eggs	58	12.9
Legume, nuts and seeds ^b^	244	54.5
Milk and milk products	226	50.5
Dietary Diversity Score		
Low	253	56
Medium	183	41
High	12	3

^a^cereals such as maize, rice, wheat, barley, other grains

^b^beans, peas, lentils, nuts, peanut

### Determinant factors of Anemia

The major determinants identified with the development of anemia were age, place of residence and status of menarche. The odds of developing anemia were almost four times more likely among late adolescents as compared to early adolescents (AOR = 3.8 95%CI = 2.3 to 8.5).Adolescents from rural areas were 3.4 times more likely to have anemia as compared to their urban counterparts (AOR = 3.4 95%CI = 1.9 to7) and adolescents those who attained menarche were two times more likely to develop anemia compared to those who did not attained menarche (AOR = 2.3 95%CI = 1.34 to 4) (Table 4).

**Table 4:** Binary and multivariable logistic regression analyses showing the impact of selected variables on Anemia in *Wayu Tuqa* distrit, Ethiopia, 2016 (*n* = 448)

Characteristics	Anemia		Crude OR	Adjusted OR
	Yes (%)	NO n (%)	(95% CI)	(95% CI)
Age				
15–19	96(5.6)	153 (34.1)	3.1 (2.67 7.1)	3.8 (2.3 8.5) *
10–14	25 (21.4)	174 (38.9)	1	
Residence				
Rural	107 (21.9)	229 (51.1)	2.8 (1.785.9)	3.4 (1.9 7.0)*
Urban	14 (3.1)	98 (21.9)	1	1
Family size				
1–5	32 (7.1)	85 (18.9)	1	–
>5	89 (19.9)	242 (54)	1(0.6 1.56)	
Status of menarche				
Attained	92 (20.5)	209 (46.7)	1.79 (1.1 2.8)	2.3 (1.3 4.2) *
Not attained	29 (6.5)	118 (26.3)	1	
Mothers educational status				
No education	61 (13.6)	146 (32.6)	1.34 (0.79 7.4)	–
Primary	37 (8)	94 (21)	1.27(.62 5.8)	
Secondary and above	26 (5.8)	84 (18.8)	1	
Dietary Diversity Score				
Low	106 (23.7)	147 (32.8)	1.44 (0.47 2.55)	**–**
Medium	73(16.3)	110 (24.6)	1.33 (0.36 13.45)	–
High	4(0.89)	8 (1.7)	1	

*denotes significance in the multivariate analysis

- Not significant

Only signifivcant varaibles are boldfaced

## Discussion

The present study was undertaken to assess the level of anemia and body mass index for age z-score (BAZ) in-school adolescent girls. Based on the adjusted hemoglobin cut-off points, the overall prevalence of anemia was 27% and the majorities suffered moderate anemia. Compared with the WHO cut-off points of 20–39%, the observed prevalence was of moderate public health significance and is consistent with the WHO estimate of adolescent girls’ anemia for developing countries which is 27% [20]. Similarly, the present finding also concurs with the Indian study findings which reported 28% of adolescent’s anemia [21] and almost similar with Kenyan study which reported 26.5% of anemia among similar participants [19].

On the other hand, compared with the national prevalence of anemia reported by Ethiopian demographic health survey (EDHS) for the year 2011, the present figure is higher (27% vs. 13.4%). This variation might be due to the type of sampled population in the present study as well as the difference in sample size [22]. Likewise, compared with the EDHS-2005 report findings, still the current finding is slightly higher than what has been reported for the nation (27% vs. 24.85%) as well as for some agro-pastoralist eco-zones namely ***Afar*** region (22.8% vs. 24.85%) where anemia is documented to be high among school going adolescent girls because of their staple diet milk which affects the bioavailability of iron [14, 18]. Although the magnitude of anemia varied, all studies indicate that anemia among adolescent in Ethiopia is of moderate public health significance.

On the contrary, when compared with some other studies done elsewhere [1, 23, 24], the present figure is lower than what has been reported in India among urban slum adolescents (27% vs. 90%). Likewise our study finding is lower than what was documented for Kurukshetra district (27% vs. 81.8%) and rural area of Raigad district (27% vs. 61%) of India. Such wide discrepancies might be attributed to the method of measuring hemoglobin, socioeconomic as well as the ethnic variation. In the present study, HemoCue was used while in the three aforementioned Indian studies, Sahli-Hellige method was used for the determination of hemoglobin which is prone to personal observation biases and might have inflated the result to some extent among others.

In this study, the prevalence of anemia was significantly higher among late adolescent age groups than the younger ones and attributed to menstrual blood loses which imposes extra demand for iron. This finding is similar with study conducted in Caste Community of Punjab, in which a positive correlation was found between age and Anemia [25]. A significantly higher prevalence of anemia was found among adolescent who were from rural areas. Adolescent from rural areas were almost four times more likely to be anemic than their urban counterpart.

This could be due to the reason that girls from rural areas might have lack of information about adequate nutrition and economic factors.

In this study, about one third (33%) of adolescents were thin or underweight. When compared with the recent report, from the same Oromia regional state, for instance, for Adama city (33% vs. 21%), and Chiro town (33% vs. 24.4) it was higher [22, 26, 27]. Nevertheless, compared with Mekele city, from the North Ethiopia, it is slightly lower (33% vs. 37.8%) [28].The observed differences could be due to variation in the socio demographic and economic characteristics of the communities [22, 26–28]. On the other hand, the proportion of overweight and obesity which was 3.6 and 1.4%, respectively were not different from previous study reports documented in ***Adama*** for overweight (3.6% vs. 3.3%) and obesity (1.4% vs. 1.0%).

In terms of dietary diversity score (DDS), more than half of them had low diversity score while high DDS was observed only in 3% of them. Compared with some previous study reports for the same regional states, the present finding was higher than what has been reported from ***Chiro*** (56.0% vs. 44.3%) and ***Adama*** (56.0% vs. 41.2%) cities. This difference need to be explained cautiously since the cities are from the same Oromia regional states with similar culture though one may expect some socio-economic difference and warrants more studies [26, 27].

The present study showed no significant association between anemia and dietary diversity score. This finding is in line with study conducted in Ghana, in which no significant association between dietary diversity and the prevalence of anemia was observed [29, 30]. This apparent lack of association might be explained by the fact that other factors other than dietary intake might have contribute to the risk of anemia. Further studies with advanced dietary assessment and laboratory methods are recommended to investigate the causes of anemia.

### Strength and limitations

We used large samples with an appropriate BMI cut off point recommended for adolescents and has shed light on the magnitude of anemia as well as thinness among in-school adolescents and make the study first of its kind in the communities. Absence of quantitative dietary intakes and failure to measure micronutrients status like serum iron, folate and vit-B12 levels due to logistic issues however were among some of the limitations in this study.

## Conclusion

The prevalence of anemia among adolescent girls was a moderate public health problem. To improve the prevailing nutritional problem, there must be inter-sectorial collaboration among health sectors and education sectors in providing nutritional education and counseling based on age and menarche status.

## Additional file

Additional file1: Questionnaire of Study- English Version (DOCX 30 kb)

## Data Availability

All the data supporting our findings are available from the corresponding author on reasonable request.

## Abbreviations

AOR: Adjusted Odds Ratio
BAZ: BMI for age Z-score
BMI: Body Mass Index
CI: Confidence Interval
COR: Crude odds Ratio
DDS: Dietary Diversiry Score
EDHS: Ethiopian Demographic and Health Survey
Hb: Hemoglobin
IDA: IronDefiencyAnemia
SD: StandardDeviation
SES: Socio-Economic Status
UNICEF: United Nation Children’s Fund
WHO: World Health Organization
