# Supplementary material for: Anemia and its determinant of in-school adolescent girls from rural Ethiopia: a school based cross-sectional study
Source: BMC Womens Health. 2019 Jul 17;19:98. doi: 10.1186/s12905-019-0791-5 (PMC6637513; doi:10.1186/s12905-019-0791-5)
Supplement: Supplementary file 1 — Questionnaire of Study- English Version (DOCX 30 kb) [file 12905_2019_791_MOESM1_ESM.docx]

Additional File1: Questionnaire of Study- English Version

| Questionnaire for study of prevalence of anemia and associated factors among adolescent girls of (10-19) years of age in wayu tuka district ,Oromia  Questionnaire Identification Number _____________________  Woreda: ____________________________________________  Kebele _____________________________________________  Name of School: _____________________________________  Date Of Interview: \|__\|__\|day \|__\|__\|month \|___\|__\|__\|___\|year  Time of Start data collection: : \|__\|__ \|hour : \|__\|__\|minute    End: \|__\|__\| hour : \|__\|__\|minute  Result \|__\|  Name of the data collector ______________________ Signature_________________  Checked By Supervisor: Name ___________________  Signature ________________  Date __________________    Result  1.compledeted  2.in completed  3.partially completed  4.other (specify) |
| --- |

| **Sr. No** | **Questions** | | ***Circle the respondent’s choice from the given alternatives*** | | **Code** | | **skip** | | |
| --- | --- | --- | --- | --- | --- | --- | --- | --- | --- |
| **Part one: know I will ask you about your Socio-demographic and Economic characteristic** | | | | | | | | | |
| **101** | | What is your kebele | _______________ | |  |  | | | |
| **102** | | What is your religion? | Orthodox……………………………1  Muslim………………………….….2  Protestant……………………………3  Catholic…………………………….4  Others, (specify) ……………….…96 | |  |  | | | |
| **103** | | To which ethnic group do you belong? | Oromo………………………………1  Amhara……………………………..2  Gurage………………………………3  Tigre ………………………………..4  Others, (specify) …………………96 | |  |  | | | |
| **104** | | Age of the adolescent? (Enter number) | ________ Years.  DOB _____/______/______ | |  |  | | | |
| **105** | | What is your grade | ___________ | |  |  | | | |
| **106** | | Marital status | single/un married……………………1  Married……………………………2  divorced……………………………3  widowed……………………………4  Other specific……………………..96 | |  |  | | | |
| **107** | | Do you have a child(children) (fill 00 if no child) | ___________________ | |  |  | | | |
| **108** | | What is your father’s/ guardian occupation? | Farmer……………………………1  Government/private employee-…….2  Merchant……………………………3  Daily labourer………………………4  Unemployed ………………………5  Others, (specify…………………96 | |  |  | | | |
| **109** | | What is your mother’s/ guardian occupation? | Housewife…………………………1  Government/private employee……..2  Farmer………………………………3  Merchant …………………………..4  Daily labourer ……………………..5  Others, (specify) …………………96 | |  |  | | | |
| **110** | | What is your father’s/ guardian educational status? | Illiterate ……………………………1  Read and write only ……………….2  Primary (1-8) ………………………3  Secondary (9-12) …………………..4  Diploma and above ………………5 | |  |  | | | |
| **111** | | What is your mothers/ guardian educational status? | Illiterate……………………………1  Read and write only ………………..2  Primary (1-8)…………………….…3  Secondary (9-12) …………………..4  Diploma and above ………………5 | |  | | |  | |
| **112** | | How many individuals live in the House | In number_________ | |  | | |  | |
| **113** | | What is the main source of water for drinking purposes? | Piped water/supply water  Piped inside dwelling 11  Piped to yard/plot 12  Public tap 13  Water from spring  Protected well/spring 21  Unprotected well/spring 22  Water from Dug well  Protected well 31  Unprotected well 32  Water form borehole  Borehole in yard/plot 41  Public boehole 42  Surface water  Pond/lake/River/stream/spring/Dam 51  Rain water 61  Other (specify) 99 | |  | | |  | |
| **114** | | What is the main source of water for washing and other purposes? | Piped water/supply water  Piped inside dwelling 11  Piped to yard/plot 12  Public tap 13  Water from spring  Protected well/spring 21  Unprotected well/spring 22  Water from Dug well  Protected well 31  Unprotected well 32  Water form borehole  Borehole in yard/plot 41  Public borehole 42  Surface water  Pond/lake/River/stream/spring/Dam 51  Rain water 61  Other (specify) 96 | |  | | |  | |
| **115** | | What kind of toilet facility do members of your household usually use | Flush toilet  Flush to Piped sewer system ………11  Flush to septic tank 12  Flush to Pit latrine ………………..13  Flush to somewhere else 14  Flush , Don’t know where 15  Pit latrine  Traditional pit toilet 21  Pit latrine with slab 22  Pit latrine with without slab 23  Ventilated improved pit latrine 24  No facility/bush/field………………….31  Other (specify)_________................96 | |  | | |  | |
| **116** | | What type of fuel does your household mainly use | Electricity . . . . . . . . . . . . . . . . . . . . . . . .1  Natural gas . . . . . . . . . . . . . . . . . . . . . . .2  Biogas . . . . . . . . . . . . . . . . . . . . . . . . . . 3  Kerosene . . . . . . . . . . . . . . . . . . . . . . . . 4  Charcoal . . . . . . . . . . . . . . . . . . . . . . . . .5  Wood . . . . . . . . . . . . . . . . . . . . . . . . . . . 6  Straw/shrubs/grass . . . . . . . . . . . . . …...7  Agricultural crop . . . . . . . . . . . . . . . . ....8  Animal dung . . . . . . . . . . . . . . . . . . . . . .9  Other specific………………………….96 | |  | | |  | |
| **117** | | Does your household have | Electricity/solar-----------------------------1  Watch/clock----------------------------------2  Radio------------------------------------------3  Television -----------------------------------4  Mobile telephone ---------------------------5  Non-mobile telephone----------------------6  Refrigerator ---------------------------------7  Table -----------------------------------------8  Bed with cotton/sponge/spring mattress-9  Electric mitad-----------------------------10  kerosene lamp/pressure lamp------------11  None --------------------------------------12 | |  | | |  | |
| **118** | | Will you please describe your family’s household living structure? | We rent a room……………………………1  We rent an apartment…………………… 2  We rent a house…………………………..3  We rent part of a house …………………4  We live in a dormitory …………………5  We live in an apartment that we own…6  We live in a house that we own………… 7  We live in part of a house that we own…8  Other (specify) ………………….. 96 | |  | | |  | |
| **119** | | Does any member of this household own | A bicycle ……………………………….1  A motorcycle or motor scooter…………2  An animal-drawn cart………………......3  A car or truck……………………...........4 None…………………………………….5 | |  | | |  | |
| **120** | | Does your household own any livestock, herd or farm animal? | Yes ………………………………..1  No ………………………………...2 | |  | | |  | |
| **121** | | If yes to Q14, how many(if none, enter '00'.) | Cows/oxen/bulls………………. \|__\|__\|  Horses\donkeys\ mules……….\|__\|__\|  Goats ………………………… .\|__\|__\|  Sheep…………………………. \|__\|__\|  Chickens……………………… \|__\|__\|  Beehives……………………….\|__\|__\| | | ----123 | | |  | |
|  | | Part Two Dietary intake and menstrual history | | | | | | | |
| 201 | | The reason for  skipping meals | No appetite…………………………1  Difficulty to digestion ……………...2  To control weight………………...3…  No time……………………………4  Just habit…………………………5  Other specific……………………..96 | | -205 | | |  | |
| 202 | | Status of menarche | Attained……………………………1  Not attained…………………………0 | |  | | |  | |
| 204 | | Age at onset of menses | **___________** | |  | | |  |  |
| 205 | | History of excessive menstrual bleeding |  | |  | | |  | |
| 206 | | Duration of blood flow | _______days | | 305 | | |  | |
| 301 | | Have you used anti malaria in last month | ___________ | |  | | |  | |
| 302 | | Walking barefoot at school and home | Yes …………………………… 1  No………………………………0 |  | | | |  | |
| 303 | | Do you smoke cigarettes | Yes …………………………… 1  No………………………………0 | |  | | |  | |
| 4 | | Part 4 knowledge assessment of Anemia | | | | | | | |
| 401 | | Have you heard about the term anemia? | Yes …………………………… 1  No………………………………0 | |  | | |  | |
| 402 | | Do you know any signs and symptoms of anemia? ( **More than one answer possible**) | Fatigue 1  Weakness 2  Dizziness 3  Headache 4  Numbness or coldness in your hands and  Feet 5  Pale skin 6  Chest pain 7  Irritability …………………………………8  Not doing well at school or work…..........9  Don’t Know about Anemia ….......88 | |  | | |  | |
| 403 | | What do you think are the causes of “anemia” in adolescents?  ( **More than one answer possible**) | Loss of excess blood...............................1  Menstruation………………………….......2  Deficiency of Iron in foods………………3  Deficiency of vitamin C in foods……….4  Other(Specify)………………………….96  Don’t Know……………………………..88 | |  | | |  | |
| 404 | | Which food items are good to prevent anemia?  ( **More than one answer possible**) | Green leafy vegetable……………………1  Non vegetarian diet……………………….2  Fruits……………………………………….3  Injera ………………………………………4  Other(Specify)……………………………96  Don’t Know………………………………88 | |  | | |  | |
| 404 | | Which food items are good to prevent anemia?  ( **More than one answer possible**) | Green leafy vegetable……………………1  Non vegetarian diet……………………….2  Fruits……………………………………….3  Injera ………………………………………4  Other(Specify)……………………………96  Don’t Know………………………………88 | |  | | |  | |

| **Part Five:** Dietary Diversity Questionnaire For Adolescents  Now I would like to ask you about the type of foods that you ate yesterday during the day and at night. Please describe the foods (meals and snacks) that you ate yesterday during the day and night, whether at home or outside the home. Start with the first food eaten in the morning. | | |
| --- | --- | --- |
| **Code** | **Examples** | **Response** |
| **501** | Any bread, rice, pasta, biscuits, or any other foods made from millet, sorghum, maize, rice, wheat? | Yes……..1  No………0 |
| **502** | \| Potatoes, sweet potato or any other food made from roots or tubers? \| \| --- \| | Yes……..  No………0 |
| **503** | pepper, carrot,cabbage | Yes……..1  No………0 |
| **504** | Banana, orange papaya | Yes……..1  No………0 |
| **505** | Meat (beef, lamb, goat, chicken | Yes……..1  No………2 |
| **506** | Any egg | Yes……..1  No………0 |
| **507** | Fish and other seafood | Yes……..1  No………0 |
| **508** | Any foods made from beans, peas, lentils, or nuts? | Yes……..1  No………0 |
| **509** | Any cheese, yogurt, milk or other milk products? | Yes……..1  No………0 |
| **510** | Any foods made with oil, fat, or butter? | Yes……..1  No………0 |
| **511** | Any Sugar, honey, sweetened soda or sugary foods such as chocolates, sweets or candies | Yes……..1  No………0 |
| **512** | Any Tea, coffee, salt | Yes……..1  No………0 |

| **Part six Hemoglobin weight and height Measurement** | | |
| --- | --- | --- |
| 601 | Height | \|___\|.\|___\|___\|cm |
| 602 | Weight | \|___\|___\|.\|___\|___\|kg |
| 603 | Heamogobin | \|___\|___\|.\|___\| g\dl |

**Thank you for your participation!!**
